# 

**Published:** 1894-03-03

**Authors:** 


**Notice to Advertisers and Subscribers.**

All Advertisements, Orders for Copies of THE HOSPITAL, and Business Communications should be addressed to The Publisher, *not to the Editor*, THE HOSPITAL, 428, Strand, W.C.

The Cost of Subscription, post free, is as follows:—Per week, 2½d.; per quarter, 2s. 8d.; per half-year, 5s. 3d.; per annum, 10s. 6d. Foreign:—Per Annum, 12s. 8d.; payable, in all cases, in advance.

**OUR NEW OFFICES.**

428, Strand, W.C., will henceforth be the Office of this Journal.

**Notices of Births, Deaths, and Marriages** are inserted in this column at a charge of 2s. 6d. for an announcement not exceeding four lines.

### NOTICE TO CORRESPONDENTS.

All MS., Letters, Books for Review, and other matters intended for the Editor should be addressed THE EDITOR, THE LODGE, PORCHESTER SQUARE, LONDON, W.

The Editor cannot undertake to return rejected MS., even when accompanied by stamped directed envelope.

**"Burdett's Hospital Annual," 1894.**

Fifth year of publication. 700 pp. Boards, gilt lettering. A most complete guide to British, Colonial, Indian, and American Hospitals, Dispensaries, Nursing and Convalescent Institutions, and Asylums. Price 5s. Published by THE SCIENTIFIC PRESS (Limited), 428, Strand, W.C.

**NOTICE.—The Index for Vol. XIV. (ending Sept. 30th, 1893) is on sale at the Office of this Journal, price 2½d., post free.**

### Books Received.

A. and C. Black.

"The Curb of Honour." By Betham Edwards.

Swan Sonnenschein and Co.

"A Student's Text Book of Botany" (first half). By Sydney Vines, M.A.

### Medical Vacancies and Appointments.

### APPOINTMENTS.

J. N. Bredin, L.R.C.S.I., L.K.Q.C.P.I., L.S.A., appointed Medical Officer to the Staff, Great Eastern Hotel, Liverpool Street. J. W. Browne, B.A., M.D., M.R.C.S.Eng., appointed an Examiner in Ophthalmology to the Royal University of Ireland. J. W. Byers, M.D., reappointed Examiner in Obstetric Medicine, Gynæcology, and Diseases of Children in the Royal University of Ireland. H. Latham, M.B., C.M.Edin., appointed Junior House Surgeon at the Stockport Infirmary. F. C. Robinson, M.R.C.S. Eng., L.R.C.P.Lond., appointed Fifth Assistant Medical Officer to the London County Asylum, Colney Hatch. A. E. Rook, L.R.C.P.Lond., M.R.C.S.Eng., appointed Medical Officer to the Eastbourne Union. W. D. Wood, L.R.C.P., L.R.C.S.Edin., appointed Medical Officer to the Bicester Local Board. E. G. Younger, M.D.Brux., M.R.C.P.Lond., appointed Physician to the Finsbury Dispensary.

### VACANCIES.

### Resident Medical Officers.

Belgrave Hospital for Children, 77 and 79, Gloucester Street, Pimlico, S.W. House Surgeon. Board, lodging, fuel, and light provided. Applications to Percy Gates, Honorary Secretary, by March 7th.

City of London Hospital for Diseases of the Chest, Victoria Park, E. House Physician. Board, residence, and allowance for washing. Appointment for six months. Applications to T. Storrar-Smith, Secretary, by March 8th.

City of London Hospital for Diseases of the Chest, Victoria Park, E. Resident Medical Officer. Salary £100 per annum, with board, &c. Applications to the Secretary, T. Storrar-Smith.

Clayton Hospital and Wakefield Dispensary. House Surgeon (Junior); unmarried. Honorarium £40 per annum. Applications and testimonials to the Honorary Secretary by March 6th.

County Asylum, Prestwich, Manchester. Assistant Medical Officer; unmarried. Salary commencing at £100 per annum, increasing to £200 by successive yearly increments of £25, with apartments, board, attendance, and washing. Applications to the Superintendent.

County Asylum, Rainhill, Liverpool. Assistant Medical Officer; unmarried, and not more than 30 years of age. Salary £100 a year, with prospect of an annual rise of £25 up to £200, with further increase according to promotion, together with furnished apartments, board, attendance, and washing. Applications and testimonials to the Medical Superintendent.

Female Lock Hospital, Harrow Road, W. Assistant House Surgeon. Board and lodging, but no salary. Appointment for twelve months. Applications and testimonials to the Secretary.

French Hospital and Dispensary, 172, Shaftesbury Avenue, W.C. Resident Medical Officer; must speak French. Salary £80 per annum, with board, furnished rooms, and attendance. Applications and testimonials to the Secretary, F. Sord, by March 1st.

Joint Counties Lunatic Asylum, Carmarthen. Medical Superintendent. Salary £500 per annum, with unfurnished house, garden, produce, fire, light, and washing. Applications and testimonials to be forwarded to W. Morgan Griffiths, Solicitor, Carmarthen, by March 24th.

London Temperance Hospital, Hampstead Road, N.W. Assistant Resident Medical Officer. Appointment for six months. No salary, but residence in the hospital, board and washing, and an honorarium of five guineas. Applications and testimonials to E. Witson Taylor, Secretary, by March 8th.

Roxburgh District Asylum, Melrose, N.B. Assistant Medical Officer. Salary £100 per annum, with board, lodging, and washing. Applications and testimonials to Dr. Carlyle Johnstone, Medical Superintendent.

Royal Surrey County Hospital, Guildford. House Surgeon. Salary £80 per annum, with board, lodging, and laundry. Applications to the Honorary Secretary by March 10th.

### Non-Resident Medical Officers.

Bacteriological Institute, Cape Colony. Medical Assistant. Salary £350 per annum. Successful candidate will be provided with a free passage (first class) to the colony. Apply by letter to Mr. Charles Loudon, W.S., 54, Queen Street, Edinburgh.

City Orthopædic Hospital, Hatton Garden. Honorary Surgeon; must be a Fellow of the Royal College of Surgeons of England or of Edinburgh. Also Two Honorary Assistant Surgeons; must be either Fellows or Members of the Royal College of Surgeons of England or of Edinburgh. Applications, addressed to the Committee at the Hospital, by March 12th.

County of Northumberland. Medical Officer of Health. Salary £500 per annum, with travelling expenses. Appointment for three years. Applications and testimonials, endorsed "Medical Officer," to C. D. Forster, Clerk to the Council, by March 24th.

Dental Hospital of London, Leicester Square, W.C. Assistant Dental Surgeon. Applications to J. Francis Pink, Secretary, by March 12th.

Owens College, Manchester. Professor of Zoology. Applications to the Council of the College, under cover to the Registrar, by April 3rd.

### The Hospital Advertiser.

### MEDICAL APPOINTMENTS.

**ROYAL SURREY COUNTY HOSPITAL, GUILDFORD.**

